# Dual Substance Use of Electronic Cigarettes and Alcohol

**DOI:** 10.3389/fphys.2020.593803

**Published:** 2020-11-02

**Authors:** Tanner J. Wetzel, Todd A. Wyatt

**Affiliations:** ^1^Department of Environmental, Agricultural and Occupational Health, University of Nebraska Medical Center, Omaha, NE, United States; ^2^Pulmonary, Critical Care, and Sleep, University of Nebraska Medical Center, Omaha, NE, United States; ^3^VA Nebraska-Western Iowa Health Care System, Omaha, NE, United States

**Keywords:** alcohol misuse, eCIG, vaping, ethanol, polysubstance use

## Abstract

Electronic cigarettes (ECs) are a modern nicotine delivery system that rapidly grew in widespread use, particularly in younger populations. Given the long history of the comorbidity of alcohol and nicotine use, the rising prevalence of ECs raises the question as to their role in the consumption of alcohol. Of the numerous models of ECs available, JUUL is the most popular. This narrative review aims to determine current trends in literature regarding the relationship between EC and alcohol dual use, as well as hypothesize potential pathogenic tissue damage and summarize areas for future study, including second-hand vapor exposure and calling for standardization among studies. In summary, EC users are more likely to participate in hazardous drinking and are at higher risk for alcohol use disorder (AUD). We surmise the pathogenic damage of dual use may exhibit an additive effect, particularly in pathogen clearance from the lungs, increased inflammation and decreased immune response, physical damage to epithelial cells, and exacerbation of chronic obstructive pulmonary disease (COPD)-like illnesses. A better understanding of pathogenic damages is critical to understand the risks placed on dual users when exposed to respiratory pathogens, such as severe acute respiratory syndrome coronavirus 2 (SARS-CoV-2).

## Introduction

The use of traditional cigarettes is one of the largest influencers of public health, contributing to more deaths per year than HIV, illicit drug use, alcohol use, motor vehicle injuries, and firearm-related injuries combined (Centers for Disease Control and Prevention, 2018). Cigarettes were subjected to a century of medical research regarding adverse health effects and nicotine addiction, along with establishing comorbidities of additional substance use. A significant body of literature demonstrates a high comorbidity between traditional cigarette smoking and harmful levels of alcohol consumption across a wide age demographic, including adolescents and young adults (Weinberger et al., 2015; Banks et al., 2017). The use of nicotine enhances the pleasurable effects felt during alcohol consumption and increases cravings for it (Thrul et al., 2019). Nicotine, one of the major addictive chemicals in cigarettes, activates the mesolimbic pathway in the brain, releasing dopamine and reinforcing addictive behavior (Sagheddu et al., 2019). Chronic alcohol consumption affects organs throughout the body, including the brain where long-term alcohol exposure induces cellular changes in neuronal pathways related to stress, motivation, and reward. Dopamine is released during alcohol consumption from the mesolimbic pathway, reinforcing alcohol ingesting behavior (Gilpin and Koob, 2008). Significant activation of pleasure and reward pathways in the brain may suggest why greater than 80% of alcohol-dependent individuals report smoking cigarettes (Romberger and Grant, 2004), daily smokers have a 17% greater risk of relapse to alcohol abuse, and smokers have a 95% greater risk of alcohol dependence when compared with non-smokers (Weinberger et al., 2015). In addition, both daily and non-daily smoking are associated with higher levels of chronic alcohol use and binge drinking, respectively (Banks et al., 2017).

Modern advancements in nicotine delivery systems sparked the creation of electronic cigarettes (ECs). First developed in 2006, ECs rapidly grew in popularity accompanied by a commensurate decrease in traditional cigarette use (Bradford et al., 2019). Prevalence rates in adolescents have increased 46% since 2014, paralleling a 48.5 and 77.8% increase in U.S. middle and high school–aged students, respectively (Dai and Leventhal, 2019). Less risk, higher popularity, and social acceptance have been cited as factors contributing to their rapid increase in popularity (Kong et al., 2015; Gorukanti et al., 2017). The rising popularity of ECs can be seen in newly created devices, such as JUUL. In 2015, JUUL, a new retail brand of EC, emerged onto the U.S. market and quickly acquired 76% of the market by the end of 2018 (Huang et al., 2019). JUUL pods utilize a proprietary nicotine salt that closely resembles free-acid nicotine and allows for more rapid absorption and delivery of nicotine to the brain. The meteoric increase in popularity has led to the term “JUULing,” which describes the action of using a JUUL (Teitell, 2017). It has become synonymous with the term “vaping” or the action of using an EC with reference to JUUL as “the iPhone of e-cigarettes” (Radding, 2015). Despite similar or higher nicotine levels than cigarettes, 39.3% of adolescents perceived JUUL as less harmful and 29.3% believed JUUL was less addictive compared with traditional cigarettes (Russell et al., 2020). Overall, the perceived safety of EC devices, rapid growth in popularity, attractive flavors, and sleek design present a significant, unknown public health concern requiring further investigation.

Given the relative newness of ECs, there is a limited body of literature detailing the role ECs play in alcohol consumption, how inaccurate perceptions of ECs contribute to risk-taking behaviors related to alcohol consumption, and the pathogenic damages that occur during dual use. In this narrative, a background will be presented using existing alcohol, smoking, and EC studies to identify potential similarities and trends, hypothesize mechanisms of damage for future study, and identify additional areas for study related to EC and alcohol dual use.

## Trend of EC and Alcohol Dual Use

**FIGURE 1:**
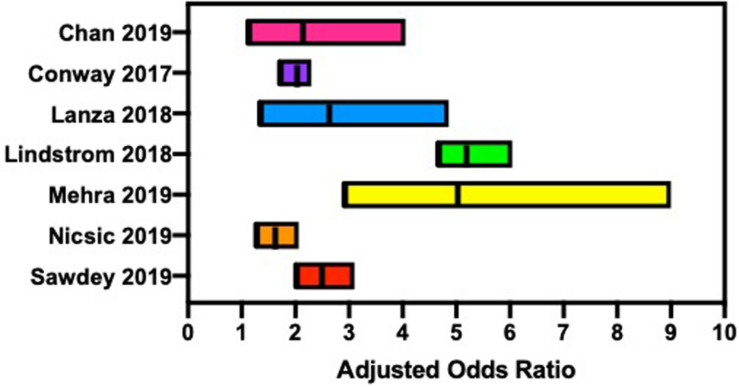
Several reports suggest that electronic cigarette users are at an increased risk for hazardous alcohol consumption, such as binge drinking and chronic alcohol consumption.

EC users have an increased risk of alcohol misuse (Figure 1), such as binge drinking or chronic use, when compared with non-EC users (Lanza and Teeter, 2018; Mehra et al., 2019). While the literature is replete for this trend with the use of traditional cigarettes and alcohol consumption, a similar public health issue may exist for EC use and requires future study. EC users reported higher alcohol use disorders identification test (AUDIT) scores, suggesting EC users are at a higher risk for an alcohol use disorder (AUD) when compared with non-users (Hershberger et al., 2016). Age of initiation was identified as a factor contributing to alcohol misuse, with a younger age of onset more likely to demonstrate lifetime alcohol use (McCabe et al., 2018). An alarming trend was identified in the increase in EC use in Canadian high school students from 2014 to 2018, along with an increase in EC and alcohol dual use from 2017 to 2018 (Zuckermann et al., 2019).

Data from the Nicotine and Other Substance Interaction Expectancy Questionnaire (NOSIE) assessed expectancies of EC and alcohol use in adults living within a community dwelling. Compared with non-EC users, EC users had significantly higher problematic alcohol use (*p* < 0.05), and combined EC and alcohol use were significantly related to problematic alcohol consumption (*p* < 0.05) (Hershberger et al., 2016). Data collected from 2,299 U.S. high school seniors examined the association between early onset of EC use and the use of other substances. A higher percentage of students who began EC use in ninth grade or earlier were more likely to report current or lifetime substance use, including alcohol (McCabe et al., 2018). Using data from the Population Assessment of Tobacco and Health (PATH) study, wave 1 (2013–2014), mental health problems related to tobacco use, including ECs, were compared with non-users. EC users were more likely to report internalization problems [adjusted odds ratio (AOR) 1.9] and substance use problems (AOR 3.4) when compared with non-users (Conway et al., 2017). These current trends may suggest a key public health concern regarding the dual use of EC and alcohol, similar to that of smoking and alcohol consumption, that needs to be investigated further.

## Effects of Nicotine and Smoking on the Lungs

Although fundamentally different, it has been suggested that the body’s response to EC use may be similar to that of cigarettes with a few unique differences (Reidel et al., 2018). To better understand the potential harmful effects of EC and alcohol on the lungs despite little empirical data, it is important to better understand the harmful effects nicotine products have on the lungs, particularly cigarette smoking. Cigarette smoking has deleterious effects on the lungs and is the leading cause of preventable death in the United States (Centers for Disease Control and Prevention, 2018). Smoking is significantly linked to lung cancer, with 80% of lung cancers in women and 90% of lung cancers in men caused by cigarette smoking (Lopez et al., 1994; Egleston et al., 2009). Besides nicotine in cigarettes, thousands of additional chemicals, such as reactive aldehydes, are shown to have carcinogenic properties (Phillips, 1996). When present in the body, these chemicals form reactive intermediates in tissues that can lead to DNA damage and cancer (Phillips, 1996). The smoke from cigarette combustion contains a high level of free radicals that can induce oxidative injury, cell membrane destruction, and inflammation within lung tissues (Munnia et al., 2006). Free radicals can induce lipid peroxidation, causing the oxidation of lipids in cell membranes, creating reactive oxygen species (ROS) and oxidative damage (Munnia et al., 2006). Additionally, ROS can interfere with the normal function of innate immune system cells, such as macrophages, neutrophils, monocytes, and eosinophils (Galvin and Franks, 2009). Macrophage -killing capacity diminishes and the recruitment of previously mentioned cells to the site of inflammation is compromised in smokers (Galvin and Franks, 2009). High levels of oxidative stress potentially play a role in the progression and exacerbation of chronic obstructive pulmonary disease (COPD) (Rahman, 2005). COPD is the third leading cause of death in the United States and, due to the long-term nature of care required, is projected to cost the U.S. health care system $49.0 billion in 2020 (Centers for Disease Control and Prevention, 2018). Given the relative newness of ECs into the market, little is known on the addictiveness of other chemicals found in ECs besides nicotine. While it is true that cigarettes contain a large number of chemicals with a variety of properties, further work is still needed to analyze the local and systemic effects of EC chemicals in the body. Secondly, the wide range of products available to the EC consumer with varying chemical compositions make standardization of testing for researchers quite difficult. Standardization of products will reduce variation between experiments and allow for a better understanding of other EC chemical effects on the body besides nicotine.

## Effects of Alcohol on the Lungs

**FIGURE 2:**
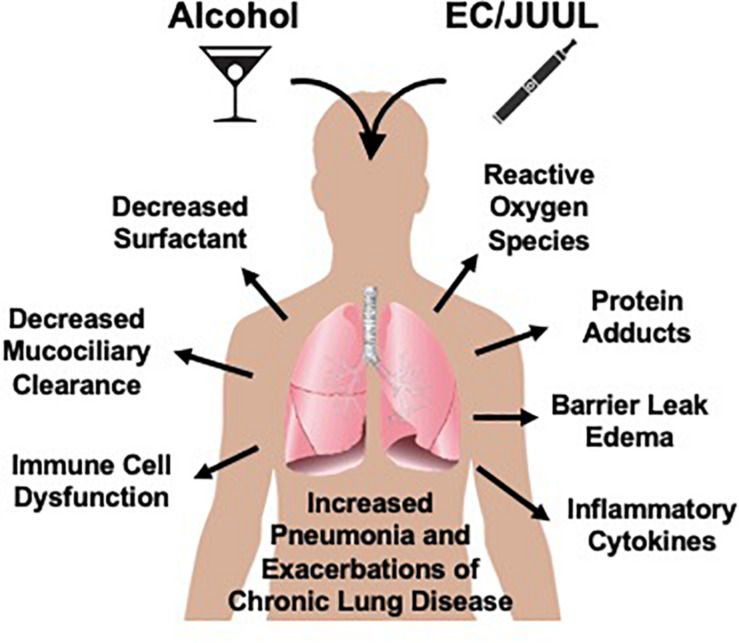
Potential for electronic cigarettes (EC) to enhance lung tissue injury. Alcohol negatively impacts the lung via direct and indirect mechanisms. As with cigarette smoking, EC generate compounds known to enhance alcohol-mediated lung injury leading to both increased risk for infections and pneumonia as well as the exacerbation of existing chronic inflammatory lung diseases such as bronchitis, emphysema, and COPD.

Moderate consumption of alcohol is typically socially acceptable and practiced by the majority of people. According to the Office of Disease Prevention and Health Promotion (ODPHP), moderate consumption is defined as two equivalent servings for men and one serving for women daily (U.S. Departments of Agriculture and Health and Human Services, 2015). However, heavy drinking, defined as more than 15 equivalent servings per week for men and 8 equivalent servings per week for women, for an extended period of time causes significant health problems throughout the body (U.S. Departments of Agriculture and Health and Human Services, 2015). The lungs are no exception (U.S. Departments of Agriculture and Health and Human Services, 2015; Figure 2). Alcohol interferes with normal innate lung immunity, particularly the physical barriers and cellular functions. Alcohol is principally metabolized by alcohol dehydrogenase in the liver after first pass via the hepatic portal vein, and during chronic alcohol consumption, by cytochrome P450 2E1 (CYP2E1) (Kaphalia and Calhoun, 2013). Up to 15% of ingested alcohol, however, is metabolized by CYP2E1 in the lungs or excreted directly via exhalation. The metabolism of alcohol produces acetaldehyde, superoxides, hydrogen radicals, and hydrogen peroxides (Aytacoglu et al., 2006), further promoting lipid peroxidation and generation of malondialdehyde (MDA) and 4-hydroxylnonenal (4-HNE) (Zheng et al., 2014). Chronic alcohol consumption is associated with bacterial pneumonia (Lujan et al., 2010), viral lung infections such as respiratory syncytial virus (RSV) (Simet and Sisson, 2015), and accumulation of fluid in the lungs due to epithelial barrier dysfunction as seen in acute respiratory distress syndrome (ARDS) (Guidot and Hart, 2005).

The first line of defense against microbial pathogens and debris in the respiratory system is the physical barriers and mucociliary transport. Chronic exposure to alcohol disrupts epithelial barrier function, allowing for paracellular leak from capillaries into the alveolar space (Guidot et al., 2000). Epithelial barriers, exposed to biologically relevant levels of alcohol for long periods, demonstrate an increased expression of sodium channels, which may serve as a counteractive measure against paracellular leak (Guidot et al., 2000). Once the tissue is inflamed, however, these mechanisms become overwhelmed and fluid accumulates within the alveolar spaces (Guidot et al., 2000). Cilia, present on the cell surface, maintain a clear airway by removing pathogens and inhaled debris out of the airway. Initial exposure to alcohol generates nitric oxide (NO) and activates a protein kinase A-dependent signaling pathway, increasing ciliary beat frequency (CBF) (Simet et al., 2013). While short-term exposure increases CBF, exposure to long-term, excessive quantities of alcohol desensitize lung airway cilia and decrease CBF when exposed to pathogens (Wyatt and Sisson, 2001). Impaired epithelial barrier and cilia function due to chronic alcohol consumption leaves the lungs more susceptible to pathogens.

Chronic alcohol also interferes with the cellular response of the innate immune system, with alveolar macrophages considered the second line of defense to invading pathogens (Rubins, 2003). Macrophages and neutrophils, key immune cells, exposed to alcohol are unable to optimally phagocytize bacteria (Joshi et al., 2009). Additionally, such macrophages have diminished release of cytokines and chemokines, which recruit other immune cells (D’Souza et al., 1996), as well as neutrophil chemoattractants to attract neutrophils to the site of inflammation (Craig et al., 2009). Thus, alcohol interferes with the recruitment of neutrophils to the lungs and increases risk for infection. Chronic alcohol also causes macrophages to undergo oxidative stress due to depletion of glutathione (GSH) stores, leading to accumulation of ROS (Yeh et al., 2007). Alcohol also interferes with granulocyte colony-stimulating factor (G-CSF), a key factor in the production of granulocytes, suppressing neutrophil production and interfering with killing potential (Bagby et al., 1998).

## Dual Effects of Smoking and Alcohol

Both the metabolism of alcohol and the inhalation of cigarette smoke contribute to significant aldehyde exposure to the lungs. Alcohol metabolism by CYP2E1 leads to the generation of superoxides, hydrogen radicals, and hydrogen peroxides (Aytacoglu et al., 2006). This, in turn, promotes lipid peroxidation and generation of MDA and 4-HNE (Zheng et al., 2014). Cigarette smoke contains high concentrations of aldehydes, such as butyraldehyde, isobutyraldehyde, propionaldehyde, acetaldehyde, formaldehyde, acrolein, propanal, and MDA (Fujioka and Shibamoto, 2006). These aldehydes further promote lipid peroxidation and generate more MDA and 4-HNE. High levels of oxidative stress from carbonyl accumulation may exacerbate COPD symptoms in dual users, with alcohol shown to be an independent risk factor for COPD (Tabak et al., 2001).

The major pathological implication of aldehyde exposure on the lungs includes oxidative stress, immune dysfunction, membrane disruption, histone modification, and mitochondrial dysfunction. Acetaldehyde, a component of cigarette smoke and metabolic product of alcohol breakdown, has epigenetic and genetic toxic effects (Shukla and Lim, 2013) and can inhibit key mitochondrial reactions and functions (Manzo-Avalos and Saavedra-Molina, 2010). Exposure to acetaldehyde induces the release of pro-inflammatory cytokines, such as tumor necrosis factor-alpha (TNF-α) and interleukin-6 (IL-6) from macrophages and IL-8 from human bronchial epithelial cells (Facchinetti et al., 2007; Moretto et al., 2009). Acetaldehyde inhibits neutrophil apoptosis, prevents neutrophil-mediated killing of pathogens, and contributes to neutrophil accumulation, resulting in a delay in resolution of inflammation (Finkelstein et al., 2005).

High levels of these reactive aldehydes form adducts on macromolecules present in the lungs, leading to an inflammatory response. Acetaldehyde and MDA form adducts on numerous nucleophilic proteins, stimulating inflammatory responses in airway epithelial cells (Wyatt et al., 2005). Acetaldehyde adducts to cilia dynein ATPase and slows CBF (Sisson et al., 1991). MDA damages macromolecules via direct adduction or indirect lipid peroxidation (Munnia et al., 2006). Formation of the malondialdehyde–acetaldehyde complex, only observed in the lungs of smokers with AUD, is stable and resists rapid degradation (McCaskill et al., 2011), leading to a protein kinase C-mediated inflammatory response (Wyatt et al., 1999). Acrolein, an aldehyde detected in cigarette smoke, increases mucin production and regulation of lung matrix metalloproteinase 9 (MMP-9), which decreases lung function in COPD patients (Deshmukh et al., 2008). It irreversibly modifies GSH stores, contributing further to oxidative stress (Yang et al., 2004). While we acknowledge this data is not explicitly testing EC and alcohol use, understanding how cigarette and alcohol use affects the lungs is important for determining testable hypotheses for potential tissue damage related to EC and alcohol use.

## Hypothesized EC and Alcohol-Mediated Lung Injury

The use of EC has potentially pathogenic health effects on the lungs. Cell types, including human fibroblasts, neutrophils, airway epithelial cells, human embryonic stem cells, and mouse neural stem cells, demonstrate morphological changes, even displaying cytotoxicity in stem cells and fibroblasts when exposed to undiluted e-liquid and flavor aldehydes, respectively (Lerner et al., 2015). EC use impairs physical barriers in the innate immune system of the lungs. EC decrease cilia, potentially as a result of ROS production (Garcia-Arcos et al., 2016; Muthumalage et al., 2019). Similarly, excessive amounts of alcohol blunt cilia responses (Sisson, 2007). In combination, EC with AUD may further slow CBF similar to that of cigarettes and alcohol (Wyatt et al., 2012), leading to an impaired ability to clear pathogens and debris from the airway. In terms of the cellular response of the innate immune system, EC vapor decreases the normal function of macrophages and neutrophils and reduces their ability to phagocytose pathogens and virus-infected cells (Sussan et al., 2015). Lipid-laden macrophages, discovered in bronchioalveolar lavage fluid (BALF) from EC users, suggest users are at risk for lipid-mediated lung injury and interfered pathogen clearance via macrophages (Eissenberg and Maziak, 2020). Lipid concentrations may be related to vaporization of propylene glycol (PG) and vegetable glycerin (VG) solvents in e-liquid. EC vapor exposure to normal human bronchial epithelium and airway epithelial cells exhibited increases in IL-6 and IL-8 production, leading to an inflammatory response and neutrophil recruitment to the lungs (Lerner et al., 2015; Garcia-Arcos et al., 2016). Neutrophils, in response to EC vapor exposure, increased the expression of proteins related to neutrophil extracellular traps (NETs), a process by which the cells excrete a net-like matrix to encompass and kill pathogens (Reidel et al., 2018). However, an increase in NET release and cell count was not observed. This may suggest that neutrophil activity is hindered by EC exposure. Pulmonary bacterial and viral clearance diminishes after exposure to EC vapor in mice (Sussan et al., 2015). Mice exposed to either EC vapor or air were exposed to *Streptococcus pneumoniae*, with EC vapor-exposed mice demonstrating a significant decrease in pulmonary bacterial clearance. After infection with influenza A, EC-exposed mice exhibited higher viral titers and enhanced viral-induced illness and mortality. Similarly, mice exposed to EC vapor increased human rhinovirus loads (Wu et al., 2014). It is widely accepted that people with AUD have higher pneumonia rates than people without AUD. In combination, EC use and AUD might demonstrate a higher rate of pneumonia than single-substance users due to significant impairment to physical and cellular defenses of lung innate immunity.

EC use is reported to cause COPD-like illnesses (Sisson, 2007; Reidel et al., 2018; Osei et al., 2020). A significant increase in the COPD-associated proteins elastase and MMP-9 was found in EC users when compared with non-users (Reidel et al., 2018). Excessive alcohol consumption is also associated with increased sputum production and cough (Sisson, 2007). Current EC use was associated with 75% higher odds of chronic bronchitis, emphysema, or COPD compared with never users (Osei et al., 2020) as well as a marked increase in mucin production (Gundavarapu et al., 2012; Garcia-Arcos et al., 2016; Javed et al., 2017). Interestingly, mucin secretion was independent of IL-13, a cytokine important in mucin regulation and appeared to be induced via nicotine receptor activation (Gundavarapu et al., 2012; Javed et al., 2017). Upregulation of CD11b and CD66b, proteins involved in the cellular adhesion and migration of macrophages and neutrophils, was detected (Higham et al., 2016). P38 MAPK, a protein kinase activated during stress and cytokine release, was also elevated (Higham et al., 2016). In addition, increased airway hyperactivity with peripheral airway flow resistance and distal airspace enlargement was detected in EC users (Lerner et al., 2015; Garcia-Arcos et al., 2016; Reidel et al., 2018). Distal airspace enlargement, often seen with the destruction of alveoli, and peripheral airway resistance may contribute to breathing difficulty and reliance on supplemental oxygen as seen in COPD patients. Damage caused by EC use may interfere with gas exchange across alveolar membranes, similar to that seen in COPD patients, leading to oxygen dependence. EC use was correlated with higher risk of emphysema and bronchitis, two conditions commonly associated with COPD (Centers for Disease Control and Prevention, 2018). In relation to alcohol, more research is needed to better understand the relationship between AUD and COPD. In moderation, alcohol functions somewhat as a bronchodilator due to the smooth muscle relaxing capabilities of nitric oxide (Sisson, 2007). Under heavy alcohol exposure, however, alcohol likely exacerbates COPD symptoms (Sisson, 2007). While it has not been investigated, AUD may exacerbate the COPD-related symptoms seen in EC users.

EC use was attributed to epithelial barrier damage in capillary endothelial cells and lung epithelial cells (Javed et al., 2017; Muthumalage et al., 2019). This damage caused leaky capillaries in the lungs, allowing for fluid accumulation unrelated to cardiac status. Epithelial damage may be the result of accumulation of ROS or particulate damage from metals found in the vapor. EC use resulted in detectable levels of ROS and free radicals, probably created during the vaporization of the e-liquid (Muthumalage et al., 2019). Similarly, EC reduced glutathione levels in cells exposed to EC vapor (Lerner et al., 2015). Glutathione plays a significant part in reducing cellular ROS and oxidative stress in cells. Decreases in glutathione levels were noted with significant increases in aldehyde detoxification proteins and oxidative stress proteins (Reidel et al., 2018).

The production of ROS by EC, paired with the reduction of cellular glutathione levels, suggests that dual users may be subjected to higher levels of oxidative stress than single-substance users. Further work is needed to determine the exact exposure levels in dual users. During alcohol metabolism by CYP450 enzymes, ROS are directly generated in the lungs. For users of EC who have an AUD, they may experience significantly higher levels of oxidative stress and damage to lung tissue when compared with single-substance users alone. For individuals who consume alcohol, a certain amount of ethanol exits the bloodstream and is exhaled. Exhaled ethanol condenses on epithelial cells, resulting in higher concentrations of ethanol in the conducting airways (Sisson, 2007). This exposure has the potential to damage cells and cause fluid to leak through tight junctions (Sisson, 2007). As a result, individuals with AUD are at an increased risk for injury-induced fluid accumulation in the lungs. In dual users, epithelial barrier damage may be further compromised, allow more fluid to accumulate in the lungs, interfere with normal gas exchange in alveoli, and contribute to an increased risk of pneumonia and ARDS.

Overall, we hypothesize that dual use of EC and alcohol may interfere with normal lung function, may contribute to the pathogenesis of COPD-like illnesses greater than single-substance users, and leave the dual user more susceptible to bacterial and viral infection. Normal innate immune responses in the lungs are altered, particularly macrophage function, decreased ciliary beating, and impaired pulmonary clearance to bacterial and viral infections. Physical barriers such as microcapillary endothelial cells and epithelial barrier cells are disrupted, causing fluid accumulation in the lungs. Nonfunctioning physical barriers and fluid accumulation in the lungs may further increase the risk of infection, decrease gas exchange, and exacerbate COPD symptoms. However, further study is required to determine the extent of the additive effects to the dual user.

## Vitamin E Acetate Lung Damage

In 2019, an outbreak of over 2,000 cases of e-cigarette, or vaping, product use-associated lung injury (EVALI) occurred across the United States, leading to 42 deaths and approximately 1,906 hospitalizations (Chatham-Stephens et al., 2019). Of those affected by EVALI, 85% reported the use of tetrahydrocannabinol (THC)-containing products within the past 3 months (Chatham-Stephens et al., 2019). A test from the CDC evaluated 29 EVALI patients and detected vitamin E acetate in BALF, providing evidence of a potential cause of injury. Vitamin E acetate is a lipid oil additive in some vaping products, particularly those containing THC, as a thickener (Wu and O’Shea, 2020). No alcohol consumption data was collected or has been reported to date on EVALI subjects. While the vaping of THC products poses a significant public health risk, it is critical to distinguish this short-term outbreak from the long-term injuries sustained by the chronic, daily use of nicotine in ECs.

## Future Areas of Interest

The relative newness of ECs as a nicotine delivery system has prevented the long-term epidemiological studies cigarettes have been subjected to. With more time, we will gain a better understanding of the addictiveness, harmful effects, and adverse health conditions that will arise as a result of chronic EC and alcohol dual use. Future study will also answer questions related to second-hand vapor exposure and the chemical composition of ECs.

Second-hand smoke is a significant public health concern related to cigarette smoking. Often, smoking and EC use occur in the same place where alcohol is consumed. This suggests second-hand vapor exposure needs to be investigated to determine the extent of unwanted exposure to bystanders in public locations. EC manufacturers claim vapor released by ECs is water vapor, thus creating no public health concern for environmental exposure. Four studies were identified that examined indoor air quality of ECs. Particulate matter (PM), particulate number count (PNC), volatile organic compounds (VOCs), polycyclic aromatic hydrocarbons (PAHs), carbonyls, and metals were found where EC use occurs (Schober et al., 2014). One study measured VOCs, nicotine, low molecular weight carbonyls, PAHs, tobacco-specific nitrosamines, and metals (O’Connell et al., 2015). Another study measured saliva and urine cotinine levels from those living in EC user homes and compared them with smoking and control homes (Ballbè et al., 2014). Nicotine deposition was measured on 10 cm^2^ areas throughout the EC and cigarette user home (Bush and Goniewicz, 2015). Overall, detectable levels of PM, PNC, 1,2-propanediol, nicotine, CO_2_, glycerin, PAHs, VOCs, and formaldehyde were present in the indoor household environment of EC users (Schober et al., 2014; O’Connell et al., 2015). Detectable levels were higher than those in control homes, yet lower than those found in the homes of smokers (Bush and Goniewicz, 2015). Levels of airborne nicotine and levels of salivary and urinary cotinine were present in individuals who did not use EC, but lived in households where EC were used, and were found to be at lower concentrations than in non-smoking individuals living in smoker households (Ballbè et al., 2014). This suggests that the vapor from ECs is not water vapor, but includes numerous chemicals found at lower levels than traditional cigarettes. Future study is required to determine if chemical concentration in second-hand vapor is biologically relevant to harm individuals consuming alcohol, or is harmful for at-risk populations, such as the elderly, immunocompromised, and pregnant women.

With a wide variety of EC devices and liquid composition, standardization and control between experiments makes comparison between studies impossible. The chemical composition of EC, both in the liquid and vapor, varied significantly depending on the EC device or liquid used (Varlet et al., 2015). PG and VG typical comprised the base ingredients, with nicotine and a variety of flavor aldehydes added as well (Varlet et al., 2015). Given its massive popularity, JUUL devices may represent an ideal model for standardization for experimentation. However, it is important to acknowledge modifications that can be added to JUUL pods, such as “hacked” pods and pods compatible with JUUL devices that contain liquid not produced by the company (LaVito, 2019). In addition, some EC liquids contained alcohol. E-liquids containing alcohol may decrease the user’s psychomotor performance and produce detectable levels of alcohol metabolites in the urine (Valentine et al., 2016). Further study is needed, however, to determine if this impairment is greater in the dual user. Flavor aldehydes represented the biggest variation in chemical composition, with numerous flavor aldehydes present such as vanillin, ethyl vanillin, benzaldehyde, cinnamaldehyde, and citral, some of which have been previously linked to lung tissue damage (Garcia-Arcos et al., 2016). While many of these aldehydes have been tested and deemed safe for oral ingestion, the effects and safety of inhalation have yet to be determined. Interestingly, one study demonstrated that PG and VG reacts with flavor aldehydes in the liquid and vapor to produce PG–aldehyde and VG–aldehyde adducts in both JUUL and EC products (Erythropel et al., 2019). These acetals had a high carryover rate from liquid to vapor during use and remained stable in physiologic pH in the lungs with a 36-h half-life. Such adducts activate cough receptors in the lungs at lower concentrations than the aldehyde alone (Erythropel et al., 2019). This study represents an important finding: reactions between ingredients in EC liquids may create stable adducts and interfere with normal lung function, demonstrating the danger of the formations of new compounds in unstable e-liquids at high temperatures found in ECs and the risk they pose to the body. Unique adduct formation, such as the formation of the malondialdehyde–acetaldehyde complex seen only in smokers with AUD (Sapkota et al., 2017), may also occur during the dual use of EC and alcohol. Future study is warranted to determine the unintended adducts formed within the dual user.

### EC + Alcohol and Coronavirus Disease 2019

Coronavirus disease 2019 (COVID-19), caused by infection of the virus severe acute respiratory syndrome coronavirus 2 (SARS-CoV-2), originated in Wuhan, China in 2019 (Wu et al., 2020). This disease quickly spread around the world, leading the World Organization (WHO) to declare COVID-19 a pandemic on March 11, 2020 (Ducharme, 2020). SARS-CoV-2 has been shown to invade cells via binding to the extracellular domain of angiotensin-converting enzyme 2 (ACE2) (Li et al., 2003; Zhou et al., 2020). Inhalation of nicotine from cigarettes has been demonstrated to increase ACE2 expression in human bronchial epithelial cells via α-7-subtype of the nicotine receptors (α7-nAChR), with a similar increase in ACE2 expression measured in patients with COPD (Brake et al., 2020; Leung et al., 2020). Studies regarding the relationship between ACE2 expression in EC-specific users are yet to be conducted, let alone studies pertaining to the effects of EC and alcohol dual use on SARS-CoV-2. Single-substance studies are starting to be published, showing higher risk of COVID-19 diagnosis in EC users; however, polysubstance use has yet to be examined (Gaiha et al., 2020). This identifies a large area of study regarding the novel coronavirus and polysubstance use. With the given aforementioned studies pertaining to individual effects of EC and alcohol on the effectiveness of lung innate immune system (Wyatt et al., 2012; Wu et al., 2014; Sussan et al., 2015), we hypothesize that dual users of EC and alcohol may be at higher risk to complications of COVID-19. However, future study regarding EC and alcohol’s effect on ACE2 when exposed to SARS-CoV-2 and comparison of symptoms between the dual user, single user, and never user are required to better understand how polysubstance use may affect an individual with COVID-19.

## Conclusion

EC and alcohol dual use is rising, with EC users more likely to participate in hazardous binge drinking, placing such users at a higher risk of AUD than non-users. With a limited body of literature compared with traditional cigarettes, a significant volume of research is still needed to better understand the long-term risks of EC and alcohol dual use (Chudomelka and Wyatt, 2020). Second-hand vapor exposure, while containing fewer chemicals than traditional cigarettes, has the potential to be harmful to vulnerable populations and requires additional study in combination with alcohol consumption. With the variety of devices and liquids available to the user, standardization for experimental testing is important. JUUL devices and liquids, given their popularity, may be the right device for such standardization. However, they too contribute challenges for research. Flavor aldehydes, nicotine, and additional chemicals formed during vaping suggest a wide variety of toxicological responses that may occur in conjunction with alcohol use that need to be investigated. The long history of comorbidity between nicotine and alcohol continues, with EC and alcohol use presenting an additional method of dual substance use yet to be explored and clearly outlines a public health issue that will not likely be diminished in the near future.

## Author Contributions

TJW and TAW agreed upon the subject matter of this review. TJW collected the review material, drafted the manuscript, and approved the final version. TAW assisted with drafting, editing, and approving the final version. Both authors contributed to the article and approved the submitted version.

## Conflict of Interest

The authors declare that the research was conducted in the absence of any commercial or financial relationships that could be construed as a potential conflict of interest.
